# Determination of Copper in Cachaça Samples Preconcentrated on Filter Paper by Portable EDXRF

**DOI:** 10.1021/acsomega.5c07845

**Published:** 2026-03-19

**Authors:** Raiane de Oliveira Araújo, Benedito Batista Farias Filho, Maria Eduarda Guida da Silva, Igor da Silva Constantino, Wilkins Oliveira de Barros, Igor José Gomes da Silva

**Affiliations:** † Programa de Pós-Graduação em Química, 67823 Universidade Federal do Piauí, Teresina, Piauí 64049-550, Brasil; ‡ Instituto Federal do Ceará, Campus Boa Viagem, Boa Viagem, Ceará 63870-000, Brasil

## Abstract

Cachaça is a distilled beverage made from sugar cane and is the most consumed spirit in Brazil. Among the inorganic elements present in this drink, copper stands out due to its potential harm to human health, which necessitates continuous quality control. In this context, the objective of this study was to develop an analytical method for the determination of copper in artisanal cachaça samples using preconcentration on a paper support and detection by portable X-ray fluorescence spectrometry (pXRF). For the analytical measurements, samples were preconcentrated on commercial filter paper using a volume of 120 μL and a temperature of 120 °C. The method was evaluated through figures of merit such as linear range (2 to 10 mg L^–1^), correlation coefficient (0.99661), detection limit (0.31 mg L^–1^), and quantification limit (0.93 mg L^–1^). The method demonstrated acceptable analytical precision, with repeatability and reproducibility values below 6.0%, and good accuracy, with recovery rates between 91.4% and 109.9%. Application of the method to different commercially available cachaça brands showed that copper concentrations did not exceed 2.5 mg L^–1^. Therefore, considering the preconcentration efficiency and the figures of merit, the method proved to be promising for copper analysis in artisanal cachaças, being fast, environmentally friendly, and low-cost, making it suitable for practical applications, such as use by small producers or for regulatory inspections.

## Graphical abstract

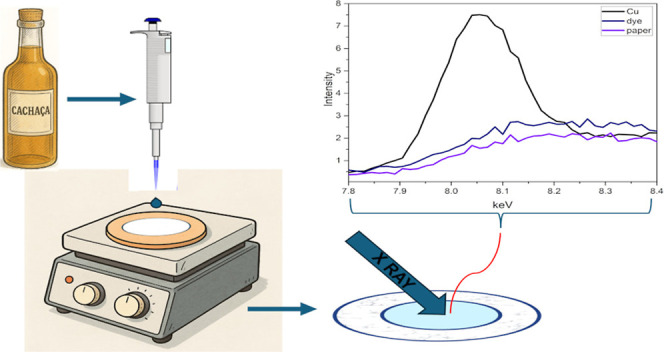

## 1 Introduction

Cachaça, a distilled beverage obtained from fermented sugar cane juice, is widely consumed in Brazil, with an alcohol content ranging from 38% to 54% at 20 °C.^1^ In addition to its cultural significance, artisanal cachaça production stands out for its unique sensory characteristics and is regulated by Ordinance No. 539/2022 of the Ministério da Agricultura e Pecuária, which defines the identity and quality standards for cachaça and aguardente.^1^

Given the high consumption of this beverage, it is essential to monitor its chemical composition to ensure consumer safety and comply with regulations that establish maximum allowable limits for contaminants. The inorganic fraction, which includes elements such as As, Cd, Ni, Zn, Mn, Pb and Cu can be influenced by factors such as sugar cane cultivation, soil contamination, distillation processes, and improper handling during production.^2,3^

Among the analytical techniques frequently employed for the determination of metals in alcoholic beverage matrices, UV–vis spectrophotometer,^4^ atomic absorption spectrometry (AAS)^5–7^ and inductively coupled plasma optical emission spectrometry (ICP-OES)^8–10^ are particularly prominent. While both methods offer high sensitivity and accuracy, they require prior sample digestion, a step that prolongs analysis time, increases the risk of volatile analyte loss, and necessitates sophisticated infrastructure and nonportable instrumentation. These constraints underscore the demand for simpler, more accessible analytical alternatives, particularly for field applications or use in laboratories with limited resources.^11^

X-ray fluorescence spectrometry (XRF) has emerged as a promising alternative due to its portability, rapid analysis time, and cost-effectiveness. The technique requires minimal sample preparation and significantly reduces the consumption of reagents and the generation of chemical waste.^12^ However, one of the principal challenges associated with XRF is the matrix effect, which can adversely affect both the sensitivity and selectivity of the analysis, particularly in complex liquid matrices.^13^

To address this limitation, various preconcentration methods have been extensively investigated.^14–17^ These procedures can be classified as either physical (e.g., evaporation, lyophilization) or chemical (e.g., coprecipitation, electrodeposition, liquid–solid, and liquid–liquid extraction) techniques.^18^ Among them, preconcentration by evaporation, although relatively slow, requires low-cost instrumentation and involves controlled heating to evaporate the matrix while minimizing analyte loss through boiling. In this context, preconcentration on paper has emerged as an innovative and effective alternative. The liquid matrix is evaporated directly onto filter paper, thereby concentrating the analytes on a solid support suitable for direct analysis. This approach not only reduces matrix interference but also retains analytical simplicity and speed, making it compatible with low-cost and portable instrumentation.^18^ Additionally, the use of paper as a support offers advantages such as ease of handling, compatibility with solid-phase analysis, and environmental sustainability, as it generates minimal waste.

Previous studies have explored paper-based or portable approaches for Cu monitoring. Santos (2012),^19^ Teixeira et al. (2012),^20^ and Meira et al. (2018)^21^ demonstrated preconcentration strategies on chromatographic paper or solid supports, but generally coupled to benchtop instruments. More recently, Maia et al. (2022)^22^ proposed a fluorescence digital image-based device using carbon dots on paper, Pessoa et al. (2017)^23^ developed a spot-test method combined with digital image capture, and Suarez et al. (2018)^24^ designed a LED-based portable photometer. While innovative, these approaches require synthesis of nanomaterials, chromogenic reagents, external light sources, or imaging devices such as smartphones or cameras.

In contrast, the present study advances this field by demonstrating, for the first time, the feasibility of using commercial low-cost filter paper as a direct preconcentration support combined with portable energy-dispersive X-ray fluorescence spectrometry (p-EDXRF). This approach eliminates the need for reagents, optical sources, or image acquisition, providing a reagent-free, environmentally friendly, and field-deployable method. Moreover, compared to other portable metal detection strategies such as electrochemical sensors, total reflection XRF (TXRF), the proposed method stands out for its operational simplicity, minimal waste generation, absence of consumables, and reduced dependence on specialized training or infrastructure. Thus, it fills an important gap between highly sensitive laboratory-based techniques and resource-demanding portable systems, offering a practical tool for both small producers and regulatory agencies.

Therefore, given the importance of ensuring the quality and standardization of cachaça production, this study proposes an analytical method that combines preconcentration on a paper support with detection by portable X-ray fluorescence (XRF). This approach addresses the classical limitations of the technique, offering a practical, accessible, and efficient alternative for the quantitative determination of copper in cachaças, with potential application by small and medium-sized producers.

## 2 Materials and Methods

### 2.1 Instrumentation

An energy-dispersive portable X-ray fluorescence spectrometer (p-EDXRF) (Thermo Fisher Scientific, Niton XL3t Ultra, Waltham, Massachusetts, USA) was employed for the determination of copper after preconcentration on filter paper (Whatman No. 40). The spectrometer is equipped with an X-ray tube featuring a silver anode, a silicon drift detector (SDD), a maximum operating voltage of 50 kV, current of 200 μA, and a power output of 2 W. For analytical measurements, the Kα emission line of copper (8.042 keV) was monitored. The instrument offers three factory calibration modes—All Geo, Mining, and Soil—which must be evaluated for suitability depending on the sample matrix, as they may influence analytical sensitivity. Additional equipment used in the procedure included an analytical balance (Denver Instrument, APX-200, Arvada, Colorado, USA), a Milli-Q water purification system (Elga, Purelab Option-Q, Woodridge, Illinois, USA), and a metal heating plate.

### 2.2 Materials and Reagents

For the analysis, a 1000 mg/L copper standard solution (Dinâmica, Indaiatuba, SP, Brazil) and absolute ethyl alcohol (99.8% v/v; Dinâmica Química Contemporânea LTDA) were used. All solutions were prepared using deionized water with a resistivity of 18.2 MΩ·cm and analytical-grade reagents. Laboratory materials and glassware were precleaned by immersion in a 10% (v/v) nitric acid solution (Dinâmica, Indaiatuba, SP, Brazil) for a minimum of 24 h. A 100 mg/L copper working solution was prepared by appropriate dilution of the 1000 mg/L stock solution.

### 2.3 Preconcentration on Filter Paper

The preconcentration procedure consisted of steps designed to increase the analyte concentration on a solid support, enabling its detection by p-EDXRF. Quantitative filter paper was used as the solid support. The preconcentration process was carried out using a heating system comprising a heating plate and a metal plate with a hollow center, which was placed directly over the heating surface and heated until reaching the predetermined temperature. Once thermal equilibrium was established, a 5 cm diameter filter paper disc was placed on the metal plate. Subsequently, standards and/or sample solutions were applied to the center of the paper in previously optimized volumes. After the final drop of solution was deposited, the system was left undisturbed to allow complete drying of the sample or standard on the paper. Once dry, the filter paper containing the preconcentrated spot was carefully removed and stored in a desiccator until analysis by p-EDXRF. The equipment is equipped with a CCD camera that allows observation of the region where the stain will be analyzed, ensuring greater reproducibility in the measurements.

### 2.4 Optimization of Sample Preparation Parameters

The sample application step on the filter paper involved the evaluation of several parameters to ensure the formation of preconcentrated spots with detectable analytical signals. For this purpose, the following variables were assessed: sample volume, heating system temperature, sample application time, presence of a colorant, and alcohol content.

#### 2.4.1 Effect of Sample Volume

To evaluate the optimal sample volume for effective preconcentration on filter paper, a 5 mg·L^–1^ copper solution containing a fixed alcohol content of 42% (v/v) was prepared. Once the filter paper reached thermal equilibrium, volumes of 30, 60, and 120 μL of the standard solution were applied to its surface. After complete drying, the papers were stored in a desiccator until analysis. All samples were prepared in triplicate.

#### 2.4.2 Effect of Temperature

The effect of the heating system temperature on the preconcentration process was evaluated at 100 °C, 120 °C, and 150 °C using a 5 mg·L^–1^ copper solution in 42% (v/v) ethanol. A volume of 120 μL of the solution was applied to the filter paper in each case. The actual temperature of the paper was continuously monitored using an infrared thermometer. All samples were prepared in triplicate.

#### 2.4.3 Evaluation of the Dye

The use of bromothymol blue dye was evaluated to facilitate the visual identification of the stains formed during the preconcentration procedure. A 0.1% (m/v) dye solution was prepared, and four drops were added to a 10 mg·L^–1^ copper solution in 42% (v/v) ethanol. The samples were applied to the filter paper and dried as previously described. All preparations were performed in triplicate.

### 2.5 Optimization of the Instrumental Parameters

Instrumental parameters of the p-EDXRF were evaluated to optimize the sensitivity of the equipment for the determination of the target analyte. Specifically, the irradiation time used during spectral acquisition and the calibration mode were systematically investigated.

#### 2.5.1 Effect of Scan Time

Stains containing 5 mg·L^–1^ of copper were prepared using a 42% (v/v) ethanol solution, with an application volume of 120 μL on the filter paper. Measurements were performed using the primary filter of the p-EDXRF instrument. Scan times of 30, 60, 90, and 120 s were evaluated, with each condition analyzed in triplicate.

#### 2.5.2 Effect of Calibration Mode

The same experimental conditions described in the previous section were used to evaluate the internal calibration modes of the spectrometer: All Geo, Soil, and Mining. For this study, the measurement time was fixed at 60 s, and all analyses were performed in triplicate.

### 2.6 Validation of the Proposed Methodology

#### 2.6.1 Analytical Curve

For the preparation of the analytical curve, a 100 mg·L^–1^ stock solution of copper was used to prepare diluted standard solutions at concentrations of 0, 2, 4, 6, 8, and 10 mg·L^–1^, with the alcohol content fixed at 42% (v/v). The analytical curves for copper determination were constructed using standards obtained by preconcentrating 120 μL of each solution on filter paper at a temperature of 120 °C. The curve was generated by correlating the emission line intensities of copper at 8.042 keV with the concentrations of the standard solutions across the evaluated range. The linearity of the analytical response and the linear regression equation of the univariate model were assessed to confirm the method’s suitability.

#### 2.6.2 Limit of Detection and Quantification

The limit of detection and quantification were calculated by eqs 1 and 2 respectively

1 $$LOD=\frac{3.3\sigma }{b}$$

2 $$LOQ=\frac{10\sigma }{b}$$

where *b* represent the slope of the analytical curve, and σ corresponds to the standard deviation, determined from the signal noise obtained by measuring ten replicates of the analytical blank.

#### 2.6.3 Precision

To evaluate the repeatability of the method, a cachaça sample was analyzed ten times using the same instrument and analyst, with preconcentrated samples prepared on the same day. For the reproducibility study, a cachaça sample was analyzed with the same instrument, but the measurements were performed by two different analysts on different days. For the preparation of the spots, 60 μL of the sample was applied, dried at 120 °C, and treated with four drops of a 0.1% m/v bromothymol blue solution. The spots were stored in a desiccator until the time of analysis.

#### 2.6.4 Accuracy

The accuracy of the method was assessed through a standard recovery test, with recovery values calculated using eq 3.

3 $$Recovery\;\left(\%\right)=\frac{\left(concentration\;of\;the\;analyte\;in\;the\;fortified\;matriz\right)}{\left(concentration\;of\;the\;analyte\;in\;standard\;solution\right)}\times 100$$

The recovery of the analytical method was evaluated by spiking samples with copper at three concentration levels: 1.0, 3.0, and 5.0 mg·L^–1^. For each concentration level, three determinations were performed, each with three replicates. To prepare the stains, 120 μL of the spiked sample was applied to the filter paper and dried at 120 °C. Additionally, four drops of a 0.1% (m/v) bromothymol blue solution were added to aid in visual identification. The stained papers were stored in a desiccator until analysis.

### 2.7 Application of the Proposed Method

To apply the proposed method, 20 cachaça samples were obtained from commercial establishments in the local market of Teresina (PI), Brazil. The selection included different brands of artisanal cachaças, ensuring the representativeness of products available in the region. After collection, the samples were stored in appropriate containers and maintained under controlled conditions until analysis.

## 3 Results and Discussion

### 3.1 Optimization of Sample Preparation Parameters

To optimize the preconcentration conditions and enhance copper retention on the filter paper, experimental variables such as sample volume, alcohol content, evaporation temperature, and the use of a revealing dye were evaluated. The results of these evaluations are presented in Figure 1.

**Figure 1:**
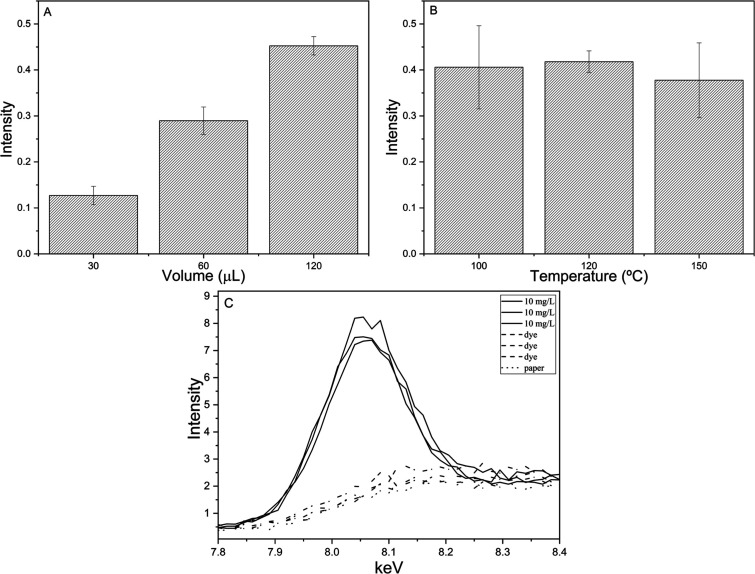
Optimization studies of the sample preparation step: (A) volume of the preconcentrated sample, (B) heating temperature for solvent evaporation, and (C) effect of the dye used to enhance visualization of the stains, ANOVA indicated no statistically significant differences between mean signal intensities (*p* > 0.05).

Increasing the sample volume applied to the center of the filter paper during the preconcentration step can enhance analytical sensitivity by depositing a greater amount of analyte on the support in multiple layers.^20^ As shown in Figure 1A, the intensities of the analytical signals obtained from preconcentrations using volumes of 30, 60, and 120 μL exhibit a linear trend, with signal intensity increasing proportionally with sample volume. This indicates an improvement in analytical efficiency with larger volumes.

The observed increase in XRF signal intensity with increasing sample volume is primarily associated with the greater absolute mass of copper deposited on the filter paper during the preconcentration step. As larger volumes are applied, successive liquid layers dry on the same region of the paper, leading to cumulative analyte accumulation within the cellulose fibers. This layered deposition increases the effective surface concentration of copper, thereby enhancing the probability of characteristic X-ray emission upon excitation. Additionally, higher deposited analyte mass improves the signal-to-noise ratio by increasing net peak intensity relative to background scattering, which is particularly relevant in portable XRF systems operating at low power. The linear relationship observed between applied volume and signal intensity indicates that self-absorption effects remain negligible within the evaluated range, confirming that the paper substrate maintains sufficient X-ray transparency. However, volumes above 120 μL resulted in excessive lateral spreading of the liquid on the paper surface, compromising spot homogeneity and spatial reproducibility.

Among the tested conditions, the use of 120 μL was selected as optimal, as it yielded significantly higher signal intensity while maintaining low standard deviations, demonstrating good reproducibility. Notably, even at the highest volume, the total sample preparation time did not exceed 15 min, confirming the method’s speed, efficiency, and suitability for routine analysis. Finally, at volumes above 120 μL, excessive spreading of the droplet on the paper was observed, compromising the homogeneity of distribution.

Another critical factor in the preconcentration stage that directly affects the analytical signal is the drying temperature of the heating plate, which influences the rate of solvent evaporation and, consequently, the total sample preparation time. Drying temperatures of 100 °C, 120 °C, and 150 °C were evaluated, as shown in Figure 1B. The results indicate no significant differences in signal intensity across the tested temperatures. However, the standard deviation served as the main criterion for selecting the optimal condition. The temperature of 120 °C exhibited the lowest variability between measurements, indicating superior reproducibility. At 100 °C, incomplete drying of the filter paper was observed, leading to extended preparation times and higher standard deviations. In contrast, at 150 °C, the paper showed initial signs of carbonization, compromising both the integrity of the sample and the effectiveness of the preconcentration process, in addition to yielding high variability.

The influence of the drying temperature on the reproducibility of the preconcentration process can be rationalized considering solvent evaporation kinetics, analyte–matrix interactions, and the physical integrity of the paper substrate. At 100 °C, the evaporation rate of the ethanol–water mixture is relatively slow, particularly due to the high ethanol content of the matrix, which leads to prolonged drying times and partial retention of residual solvent within the paper fibers. This incomplete drying favors local analyte redistribution during evaporation, resulting in less homogeneous copper deposition and higher signal variability. At 150 °C, although solvent removal is faster, the elevated temperature promotes excessive thermal stress on the cellulose matrix, causing incipient paper darkening and partial structural degradation. Such effects may alter the surface morphology and local density of the paper, negatively impacting X-ray interaction conditions and increasing signal dispersion.

In contrast, drying at 120 °C provides a balanced condition in which solvent evaporation is sufficiently rapid to prevent analyte migration, while preserving the structural integrity of the paper support. Under these conditions, copper remains stably retained within the cellulose network, leading to more homogeneous analyte distribution and improved reproducibility. Similar optimal temperature behavior has been reported in ring-oven and paper-based preconcentration techniques, where moderate heating ensures efficient solvent removal without analyte loss or substrate degradation. Therefore, 120 °C was selected as the most appropriate temperature, providing efficient drying, rapid processing, and consistent analytical performance. In addition, this temperature is safe to ensure proper deposition of copper without volatilization or decomposition, as demonstrated in previous studies using the ring-oven technique.^15,17^

To statistically support the selection of the optimal experimental conditions, one-way analysis of variance (ANOVA) was applied to compare the mean analytical signals obtained under different sample volumes and drying temperatures at a confidence level of 95% (*p* = 0.05). The results indicated that no statistically significant differences were observed among the mean intensities for the evaluated conditions (*p* > 0.05). Therefore, the selection of 120 μL and 120 °C was based primarily on the minimization of relative standard deviation (RSD) and improved reproducibility, rather than on differences in mean signal intensity.

Figure 1C presents the results of the study on the effect of adding bromothymol blue dye (0.1% m/v) on the analytical signal of copper (The 10 mg/L refers to the standard containing the dye. “Dye” corresponds to the analytical blank containing only the dye, while “Paper” refers to the analysis of the paper alone). The data show that the addition of the dye did not interfere with the analytical results, as the signal remained consistent with that of the analytical blank. The use of bromothymol blue enabled clear visualization of the region where the sample was applied, as the dye induced a color change from colorless to yellow, precisely delineating the preconcentration area. This visual marker facilitated the identification and consistent positioning of the analysis point for p-EDXRF measurements. Therefore, to improve the visibility of the preconcentrated stains and ensure accurate spot analysis, bromothymol blue was incorporated into the subsequent steps of the preconcentration procedure, contributing to the enhancement of the overall analytical methodology.

### 3.2 Evaluation of the Optimization of Instrumental Parameters

Figure 2 presents the results obtained from the optimization of instrumental parameters for analysis by p-EDXRF, conducted following the preconcentration step of the samples.

**Figure 2:**
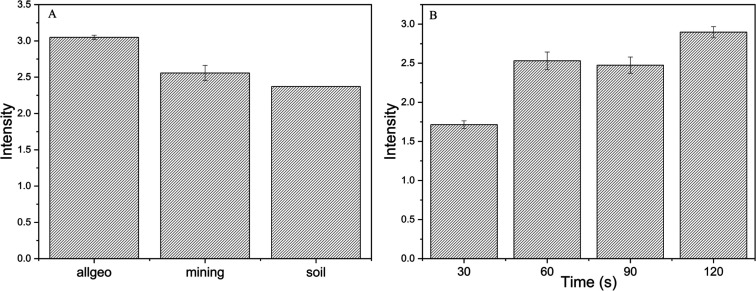
Studies of instrumental optimization (A) calibration type, (B) measurement time.

The p-EDXRF instrument offers three internal calibration modes, All Geo, Mining, and Soil, that are designed to accommodate a wide range of sample types. Each calibration must be evaluated for the specific sample matrix and target analyte, as analytical sensitivity can vary significantly. Figure 2A displays the results obtained from the analysis of a preconcentrated spot using each of these calibration modes. The All Geo calibration produced a markedly higher analytical signal compared to the Soil and Mining calibrations. This superior performance indicates enhanced sensitivity and detection efficiency for the analyte of interest, supporting the selection of the All Geo calibration as the most appropriate choice for subsequent analyses in this study.

In quantitative studies employing X-ray fluorescence spectrometry, the irradiation time is a critical parameter for achieving results with both high sensitivity and precision. As highlighted by Kalnicky and Singhvi^25^ and Parsons et al.,^26^ optimizing scan times is essential and should be tailored to the specific characteristics of each sample type. Measurement time may vary depending on the instrument, analyte, or matrix configuration. Figure 2B presents the analytical results obtained from the evaluation of measurement times of 30, 60, 90, and 120 s, aiming to identify the optimal acquisition time. The data reveal a progressive increase in the analytical signal with increasing measurement time. The 30 s interval resulted in the lowest signal and was therefore excluded from further use. A significant enhancement in signal intensity was observed at 60 s, which remained relatively stable at 90 s. At 120 s, a further notable increase in signal intensity was observed, accompanied by high precision among replicates. Based on these findings, a measurement time of 120 s was selected for all subsequent analyses to ensure optimal sensitivity and analytical reliability.

### 3.3 Validation of the Proposed Methodology

For validation of the proposed analytical method, the following parameters were evaluated: linearity, limit of detection (LOD), limit of quantification (LOQ), accuracy (recovery), and precision (reproducibility and repeatability). The analytical curve, constructed using preconcentration of 120 μL on a heating plate at 120 °C with a 42% (v/v) ethanol solution and copper concentrations ranging from 2 to 10 mg·L^–1^ (Figure 3), yielded a correlation coefficient of *r* = 0.99661 and analytical curve equation of *I* = 0.28218 + 0.54081*C* (*I* is intensity and *C* is concentration), demonstrating satisfactory linearity and robustness of the proposed method. Figure 3 still shows the raw spectra of the standards without subtraction of the analytical blank. This result confirms the method’s capability to reliably quantify copper, emphasizing the effectiveness of the preconcentration procedure in enhancing analytical signal intensity. Although the curve was extended to 10 mg·L^–1^ for evaluation of the method’s dynamic range, it is important to note that regulatory limits are more stringent, commonly set at 5 mg·L^–1^ or even 2 mg·L^–1^. Nonetheless, the method showed adequate sensitivity and accuracy at lower concentrations, ensuring compliance with existing regulatory standards.

**Figure 3:**
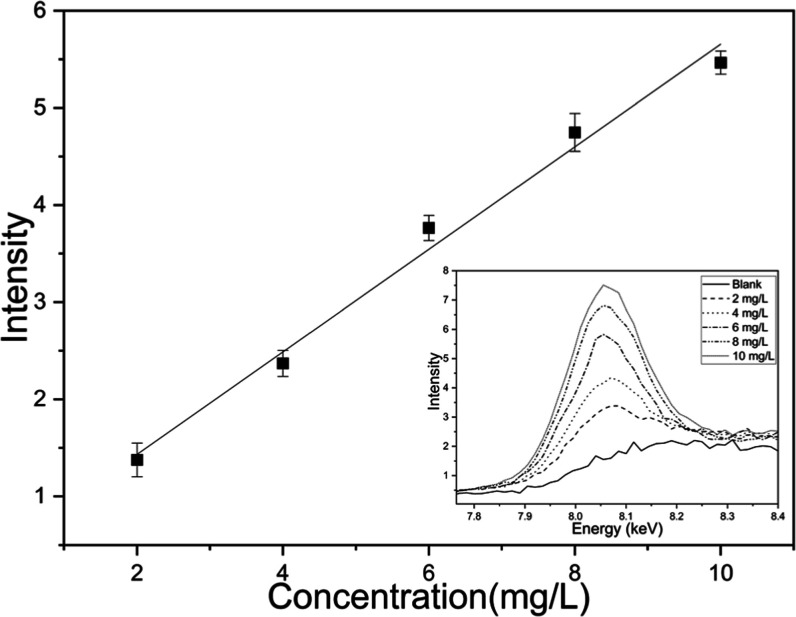
Analytical curve constructed using copper standard solutions at concentrations ranging from 2 to 10 mg·L^–1^, with preconcentration of 120 μL on a heating plate at 120 °C in a 42% (v/v) ethanol solution and raw spectra of the standards without subtraction of the analytical blank.

The maximum liquid intensity of the blank (*n* = 10) after baseline discount provided value 0.22 + 0.05 arbitrary unit. The limits of detection (LOD) and quantification (LOQ) were calculated according to eqs 1 and 2, using the standard deviation of the blank (σ) and the slope (*b*) of the analytical curve. The value of σ was obtained from ten replicate measurements of the analytical blank, resulting in σ = 0.05 (arbitrary intensity units), while the slope of the calibration curve was *b* = 0.54081 (intensity units·L·mg^–1^). Based on these parameters, LOD and LOQ values of 0.31 mg·L^–1^ and 0.93 mg·L^–1^, respectively, were obtained. Although the LOQ was estimated as 0.93 mg·L^–1^, quantitative results are considered fully validated within the linear working range of 2–10 mg·L^–1^. These values indicate satisfactory analytical performance, particularly when considering the simplicity of the procedure, the portability of the equipment, and the elimination of complex sample preparation steps. Although techniques such as flame atomic absorption spectrometry (FAAS) offer lower detection limits, they require acid digestion, as well as the use of gases and chemical reagents, which increase both the cost and complexity of the analysis.

Electroanalytical techniques, such as differential pulse anodic stripping voltammetry (DPASV), can achieve even lower detection limits, on the order of 0.05 μg·L^–1^,^27^ but require specialized laboratory infrastructure and stricter experimental control. In the context of XRF, Santos^19^ reported a LOQ of 0.047 mg·L^–1^ for copper in cachaça using a benchtop EDXRF spectrometer and preconcentration on cationic chromatographic paper. Although the method proposed in the present work yields higher detection and quantification limits, it is based on the use of a p-EDXRF instrument and common filter paper, which accounts for the reduced sensitivity. Nevertheless, the values obtained remain below the maximum limits established by Brazilian regulations, confirming the method’s applicability for monitoring copper in cachaça.

The precision of the analytical method developed in this study was evaluated through repeatability and intermediate precision tests. Repeatability was assessed by analyzing ten samples prepared under identical operating conditions, on the same day, and by the same analyst. Intermediate precision was determined by analyzing ten samples prepared on two different days by two different analysts. In both cases, the relative standard deviation (RSD) was calculated to assess the method’s repeatability and intralaboratory reproducibility. The results are presented in Figure 4.

**Figure 4:**
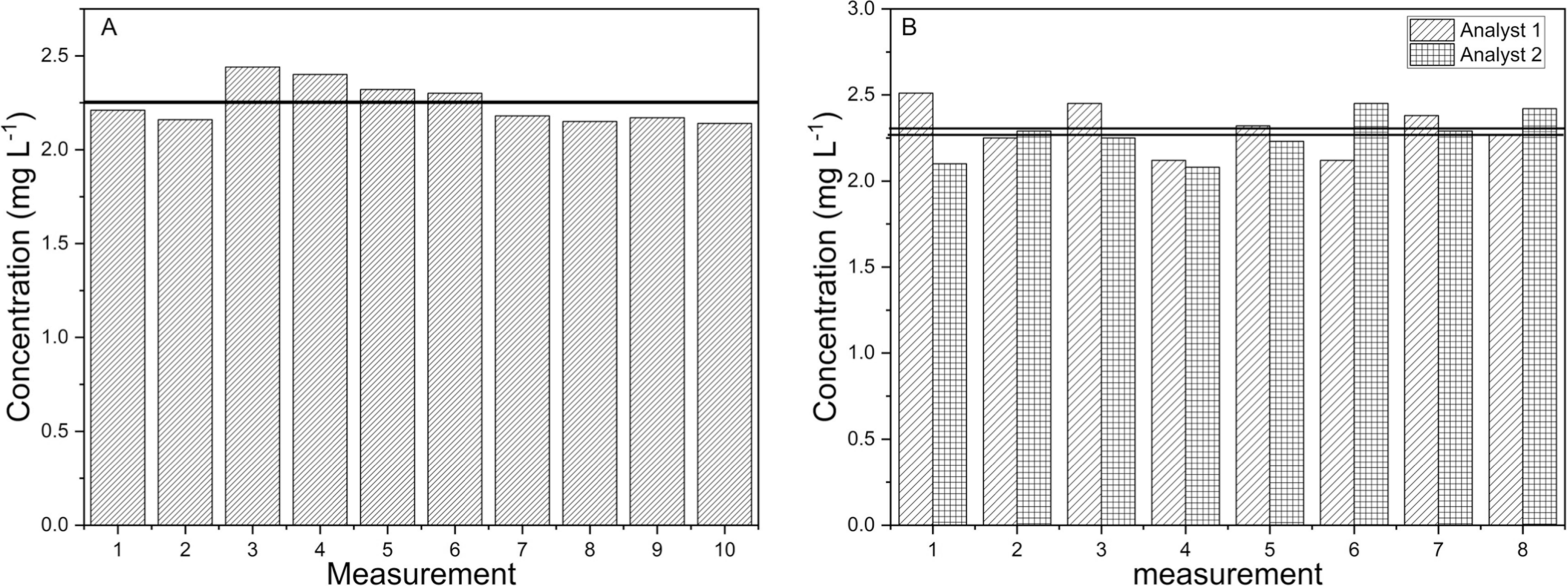
Evaluation of repeatability (A) and intermediate precision (B) in the preconcentration of a cachaça sample.

In the repeatability test, conducted under identical operating conditions (same analyst and equipment), an absolute standard deviation of 0.11 mg·L^–1^ and a relative standard deviation (RSD) of 4.89% were obtained (Figure 4A). For the intermediate precision test, which involved variation in both analyst and day of analysis, the absolute and relative standard deviations were 0.13 mg·L^–1^ and 5.84%, respectively (Figure 4B). These results indicate an acceptable level of variation for an alternative analytical method designed to prioritize simplicity, portability, and cost-effectiveness.

When comparing the results obtained in this study with data available in the literature, it is evident that conventional methods, such as graphite furnace atomic absorption spectrometry (GF AAS) or flame atomic absorption spectrometry (FAAS), typically offer greater accuracy, albeit with higher operational costs and greater procedural complexity.

In the study by Caldas et al.,^28^ GF AAS was employed for the determination of copper, lead, and arsenic in brandy, yielding RSD values of approximately 3.3% for copper, which indicates excellent reproducibility. However, this level of precision required prior acid digestion of the samples, highlighting the trade-off between analytical performance and methodological simplicity.

On the other hand, alternative methods based on energy-dispersive X-ray fluorescence (EDXRF) combined with preconcentration have shown considerable promise. Santos^19^ developed a method for the determination of copper, iron, nickel, and zinc in ethanol and cachaça using ion-exchange chromatographic paper as the preconcentration support. For copper, RSD values ranging from 2.5% to 3.1% were obtained in ethanol matrices of 50% and 96%, based on 15 consecutive determinations. The methodology did not require acid digestion and enabled direct analysis of the paper; however, the chromatographic paper used incurs a higher cost compared to standard filter paper.

In another study, Meira et al.^21^ employed energy-dispersive X-ray fluorescence spectrometry following magnetic solid-phase extraction for the determination of metals in cachaça. The method achieved an RSD of 1.5% for copper at a concentration of 0.2 mg·L^–1^. Despite its precision, the use of nanoparticles and benchtop instrumentation limits its simplicity and portability. Similarly, Ferreira et al.^27^ investigated voltammetry using carbon nanotube-modified electrodes for copper analysis in alcoholic beverages. Although the technique demonstrated high sensitivity, it requires stringent control of experimental variables, multivariate calibration, and does not offer the same potential for field analysis as methods based on portable XRF.

The recovery assay is a fundamental step in the validation of analytical methods, as it assesses the method’s accuracy by adding known amounts of the analyte to the sample and determining the proportion effectively recovered after the analytical procedure. In the present study, known concentrations of copper (1.00 mg·L^–1^, 3.00 mg·L^–1^, and 5.00 mg·L^–1^) were added to a cachaça sample with an initial copper concentration of 2.25 mg·L^–1^. The lowest recovery level (1.0 mg·L^–1^) was intentionally selected near the LOQ to evaluate method accuracy under limiting conditions, which is consistent with international validation practices for trace analysis. Recovery was evaluated at three levels, and the results are presented in Table 1.

**Table 1:** Recovery Study for Copper Determination in Cachaça Samples Using Portable X-ray Fluorescence (p-EDXRF)

sample concentration (mg/L)	concentration added (mg/L)	recovered concentration (mg/L)	recovered (%)	relative standard deviation (%)
2.25	1.00	1.05 ± 0.08	105.0 ± 8.5	8.1
	3.00	3.30 ± 0.07	109.9 ± 8.0	7.3
	5.00	4.57 ± 0.18	91.4 ± 3.5	3.8
	1.00	1.06 ± 0.09	106.0 ± 8.8	8.3
1.80	3.00	3.00 ± 0.09	100.0 ± 7.3	7.5
	5.00	4.52 ± 0.17	90.4 ± 2.7	3.0
	1.00	1.08 ± 0.07	108.0 ± 8.6	8.0
1.50	3.00	3.19 ± 0.10	106.3 ± 8.0	7.5
	5.00	4.77 ± 0.15	95.5 ± 3.3	3.5

The results indicated recovery values ranging from 90.4% to 109.9%, with relative standard deviations (RSD) below 8.1%, demonstrating good accuracy of the proposed method across the concentration range studied. According to ANVISA^29^ guidelines, analytical methods for the determination of trace elements should exhibit recovery values between 80% and 120%, with acceptable variations of up to 15% for complex matrices. These criteria were fully met by the method evaluated in this study, confirming its suitability for copper determination in cachaça samples.

In the literature, similar or even more stringent recovery values have been reported in studies employing X-ray fluorescence methodologies combined with preconcentration. Santos^19^ reported recovery rates between 92% and 99% for copper, iron, nickel, and zinc, with relative standard deviations not exceeding 5% across the studied ranges, demonstrating the effectiveness of the solid support and the good reproducibility of the method, even in matrices with high ethanol content. Likewise, Meira et al.,^21^ using EDXRF following solid-phase extraction, obtained copper recoveries ranging from 95% to 104% in cachaça samples, also with RSDs below 5%. Although these methods show a slight advantage in terms of precision, they involve more complex sample preparation procedures and rely on fixed laboratory instrumentation, which contrasts with the portability and simplicity of the method proposed in the present study. Although the standard deviation reached 8.3%, this value is acceptable under the standards established by the Brazilian regulatory,^29^ considering the inherent variability of the method, particularly because it involves a portable instrument.

To contextualize the analytical performance of the proposed method, Table 2 presents a comparative overview of representative methodologies reported in the literature for the determination of copper in cachaça and related alcoholic matrices. The comparison includes key analytical figures of merit, such as linear working range, limits of detection, and the analytical techniques employed.

**Table 2:** Comparison of Analytical Methods Reported in the Literature for Copper Determination in Cachaça Samples, Including Linear Working Range, Limits of Detection (LOD), and Analytical Techniques

reference	linear range (mg/L)	LOD (mg/L)	analytical technique
this work	2–10	0.31	portable EDXRF after preconcentration on filter paper
Meira et al. (2019)	not explicitly stated	0.032	benchtop EDXRF after magnetic solid-phase microextraction (CoFe_2_O_4_–PAN)
Cunha e Silva et al. (2004)	up to ∼2.25	0.008–0.035	TXRF after dry-ashing digestion
Caldas et al. (2009)	0–1.50	0.14	GF AAS with permanent modifiers
Rocha et al. (2008)	0.2–20.0	0.05	UV–vis spectrophotometry
Villis et al. (2018)	0.006–0.201	0.00074	voltammetry with ionic-imprinted hybrid electrode
Ferreira et al. (2020)	multivariate model	not explicitly stated	voltammetry with carbon nanotube electrode + chemometrics

In contrast, the present method demonstrates competitive performance within the concentration range relevant to regulatory control, offering advantages in terms of simplicity, portability, low cost, and minimal reagent consumption.

### 3.4 Application of the Method

Following the optimization and validation steps, the proposed method was applied to the analysis of 20 commercial cachaça samples, as presented in Table 3.

**Table 3:** Quantitative Determination of Copper in 20 Cachaça Samples Using the Proposed Method

sample	concentration (mg L^–1^)
A–L, N, O, Q, S, T	<LR^a^
M	2.1 ± 0.1
P	2.2 ± 0.1
R	2.5 ± 0.1

a Linear range.

The results indicate that only three of the analyzed samples (M, P, and R) exhibited quantifiable copper concentrations, ranging from 2.1 to 2.5 mg·L^–1^. The remaining samples presented copper levels below the linear range of the method. All quantified samples displayed copper concentrations well below the maximum limits established by regulatory standards, indicating compliance and no immediate risk to consumer health in this regard. The presence of copper in cachaça is primarily attributed to leaching from copper stills, particularly in artisanal distilleries that may not perform adequate maintenance or fail to discard the initial distillation fractions. The sample M, P, and R, with a concentration above of 2.0 mg·L^–1^, showed the highest copper content among the analyzed samples, yet remained within acceptable levels for artisanal products. Previous studies have also reported a wide variation in copper concentrations in cachaça samples available on the Brazilian market, reinforcing the importance of continued monitoring. Labanca and Glória^30^ analyzed 71 samples of cachaça and brandy marketed in Minas Gerais and reported an average copper concentration of 2.30 mg·L^–1^, with a maximum value of 12.2 mg·L^–1^, indicating that the values obtained in the present study are below the average observed at that time.^30^

Küchler and Silva,^31^ using potentiometric methods and flame atomic absorption spectrometry (FAAS), analyzed 21 samples from various brands and found concentrations ranging from <0.03 mg·L^–1^ to 5.86 mg·L^–1^, with an average around 2 mg·L^–1^; three of those samples exceeded the legal limit of 5 mg·L^–1^, which was not observed in any sample analyzed in this study. Cunha e Silva et al.^32^ employing total reflection X-ray fluorescence (TXRF), identified two samples with copper concentrations above the regulatory limit, reinforcing the variability in the quality of commercial products.

In samples presenting copper concentrations below the linear range (<LR), the absence or very low levels of copper can be attributed to several factors related to production practices and raw material handling. The use of stainless steel or mixed-material distillation systems, adequate cleaning and maintenance of copper stills, and the proper discard of the initial distillation fractions (heads) are known to significantly reduce copper leaching into the final product. Additionally, variations in fermentation conditions, contact time between the distillate and copper surfaces, and storage practices may further influence copper levels. Therefore, the occurrence of <LR values likely reflects improved manufacturing control and compliance with good production practices rather than analytical limitations of the proposed method.

In the present study, the fact that same samples exhibited copper levels below the linear range also points to good product quality with respect to copper contamination. This suggests the adoption of appropriate manufacturing practices and effective distillation control. Furthermore, it highlights the sensitivity of the proposed method in detecting very low copper concentrations, an essential requirement for ensuring food safety and meeting international export standards, which may impose even more stringent limits.

### 3.5 Method Limitations and Future Perspectives

Although the proposed method demonstrated satisfactory performance for copper determination in cachaça samples, some limitations should be acknowledged. The present study focused exclusively on copper due to its regulatory relevance and relatively high XRF sensitivity; therefore, the applicability of the preconcentration strategy to other trace metals with lower fluorescence yields or overlapping emission lines was not systematically evaluated. Regarding matrix effects, the method was validated using cachaça samples with ethanol contents close to the typical commercial range (≈38–54% v/v). Although variations in ethanol concentration may influence evaporation kinetics during the preconcentration step, no significant analytical bias was observed within this range. The potential influence of organic compounds naturally present in artisanal cachaça, such as congeners derived from fermentation and aging, is expected to be minimized by the evaporation-based preconcentration and solid support analysis. Additionally, differences between artisanal and industrial cachaça, including production scale, raw material handling, and distillation equipment, may lead to variability in matrix composition and copper levels. While the proposed method proved robust for the samples evaluated, further studies involving a broader range of matrices and production conditions would be beneficial to fully assess its general applicability.

## 4 Conclusion

The present study successfully achieved its objective of developing a simple, efficient, and low-cost analytical method for the determination of copper in cachaça samples. The proposed strategy combined portable X-ray fluorescence (p-EDXRF) with preconcentration on filter paper, effectively overcoming limitations commonly associated with matrix effects in liquid samples. Through the systematic optimization of experimental variables, including sample volume, evaporation temperature, measurement time, and calibration mode, a reproducible and sensitive operational protocol was established. The analytical performance of the proposed methodology was confirmed by the obtained figures of merit. The detection limit (0.31 mg·L^–1^) and quantification limit (0.93 mg·L^–1^) were adequate for compliance monitoring with Brazilian legislation, which sets a maximum allowable copper concentration of 5 mg·L^–1^ in distilled beverages. Precision tests yielded relative standard deviations below 6%, while recovery rates ranged from 91.4% to 109.9%, meeting the criteria established by national regulatory agencies. Application of the method to eight commercial cachaça samples revealed copper concentrations below legal limits, with five of the samples presenting levels below the method’s limit of quantification. These findings not only validate the reliability of the proposed methodology but also reflect a favorable scenario regarding copper contamination control in products from the evaluated region. Therefore, it can be concluded that the developed method represents a viable alternative for laboratories with limited infrastructure, offering particular utility for field inspections, monitoring by artisanal producers, and quality control in small-scale production units. Its operational simplicity, rapid execution, low cost, and environmentally sustainable approach constitute a significant contribution to analytical methodologies for the determination of metals in alcoholic beverages.
